# Trends and Patterns in the Public Awareness of Palliative Care, Euthanasia, and End-of-Life Decisions in 3 Central European Countries Using Big Data Analysis From Google: Retrospective Analysis

**DOI:** 10.2196/28635

**Published:** 2021-09-20

**Authors:** Matthias Huemer, Daniela Jahn-Kuch, Guenter Hofmann, Elisabeth Andritsch, Clemens Farkas, Walter Schaupp, Eva Katharina Masel, Philipp J Jost, Martin Pichler

**Affiliations:** 1 Division of Oncology with affiliated Unit of Palliative Medicine Department of Internal Medicine Medical University of Graz Graz Austria; 2 Division of Oncology Department of Internal Medicine Medical University of Graz Graz Austria; 3 Institute of Moral Theology University of Graz Graz Austria; 4 Clinical Division of Palliative Care Department of Medicine I and Comprehensive Cancer Center Medical University of Vienna Vienna Austria; 5 Department of Internal Medicine III - Hematooncology Technical University of Munich School of Medicine Technical University of Munich Munich Germany

**Keywords:** Google Trends, end-of-life decisions, assisted suicide, euthanasia, palliative care, health care policy

## Abstract

**Background:**

End-of-life decisions, specifically the provision of euthanasia and assisted suicide services, challenge traditional medical and ethical principles. Austria and Germany have decided to liberalize their laws restricting assisted suicide, thus reigniting the debate about a meaningful framework in which the practice should be embedded. Evidence of the relevance of assisted suicide and euthanasia for the general population in Germany and Austria is limited.

**Objective:**

The aim of this study is to examine whether the public awareness documented by search activities in the most frequently used search engine, Google, on the topics of *palliative care, euthanasia*, and *advance health care directives* changed with the implementation of palliative care services and new governmental regulations concerning end-of-life decisions.

**Methods:**

We searched for policies, laws, and regulations promulgated or amended in Austria, Germany, and Switzerland between 2004 and 2020 and extracted data on the search volume for each search term topic from Google Trends as a surrogate of public awareness and interest. Annual averages were analyzed using the Joinpoint Regression Program.

**Results:**

Important policy changes yielded significant changes in search trends for the investigated topics. The enactment of laws regulating advance health care directives coincided with a significant drop in the volume of searches for the topic of euthanasia in all 3 countries (Austria: −24.48%, *P*=.02; Germany: −14.95%, *P*<.001; Switzerland: −11.75%, *P*=.049). Interest in palliative care increased with the availability of care services and the implementation of laws and policies to promote palliative care (Austria: 22.69%, *P*=.01; Germany: 14.39, *P*<.001; Switzerland: 17.59%, *P*<.001). The search trends for advance health care directives showed mixed results. While interest remained steady in Austria within the study period, it increased by 3.66% (*P*<.001) in Switzerland and decreased by 2.85% (*P*<.001) in Germany.

**Conclusions:**

Our results demonstrate that legal measures securing patients’ autonomy at the end of life may lower the search activities for topics related to euthanasia and assisted suicide. Palliative care may be a meaningful way to raise awareness of the different options for end-of-life care and to guide patients in their decision-making process regarding the same.

## Introduction

### Background

How and to what extent a person may wish to be cared for when terminally ill and shortly before death should depend, without a doubt, solely on the expressed will of the concerned individual. Freedom of choice also includes the personal right to choose death over life [1]. In the case of advance health care directives, the right to a nontreatment decision and the consensual withdrawal of potentially curative treatments are protected by regulating acts in most Western countries [2]. Furthermore, suicide is not considered a crime in such places [2]. However, there is an ongoing debate on the extent to which a second person should assist a person in willingly dying [3]. Legal regulations concerning euthanasia and assisted suicide vary considerably among European countries. Although the Netherlands, Luxembourg, and Belgium allow euthanasia and assisted suicide under specific circumstances, Switzerland has more liberal legislation concerning assisted suicide but not euthanasia. Other countries in Europe strictly prohibit a death that involves a second person [2].

Principally, assisted suicide is not prohibited in Germany, as suicide per se is not considered a crime, but its commercial practice was restricted in 2015 [2]. On the other hand, Austria considers assisted suicide a crime per se and prohibits its practice entirely [2]. However, in 2020, the debate about end-of-life decisions was reignited in Germany and Austria after the constitutional courts in both countries declared the restriction of assisted suicide unconstitutional [4,5]. Both countries are currently in the process of liberalizing their legal regulations concerning this topic. However, voluntary death on demand remains prohibited in both countries [4,5].

Palliative care is often considered an alternative and sometimes even contradictory to the active termination of a patient’s life upon voluntary request [6]. The traditional aim of palliative care is to improve the quality of life of a severely ill person and to neither shorten nor unnecessarily prolong that life [7]. Hence, the European Association of Palliative Care (EAPC) dissociated itself from implementing euthanasia and assisted suicide in the context of palliative care in a recently published white paper [6]. Furthermore, the preventive effect of palliative care on the desire to hasten death and the request for euthanasia or assisted suicide has been repeatedly proposed but never confirmed [8,9]. It is more likely that palliative care may act as a filter for patients to differentiate between those who seek euthanasia and assisted suicide because of intrinsic reasons and those who have reactive suicidal thoughts because of severe temporary suffering [8,10]. The desire to die may be either a true conviction of euthanasia being the only acceptable option for the patient or a way to express suffering without the explicit wish to hasten death actively [10]. Suffering could be alleviated through palliative care, potentially leading to a lower desire to die in some patients [11]. However, it seems that palliative care does not prevent the request for euthanasia in patients who are already truly convinced to commit assisted suicide or euthanasia [8]. Hence, patients expressing a desire to die should be referred to palliative care to explore the underlying reasons resulting in a desire to hasten death before granting a request for assisted suicide or euthanasia [8-11].

Surveys among the general public in Germany and Austria show an increasing acceptance of euthanasia and assisted suicide [12-17]. However, these surveys often reflect only a general opinion toward the topic and rarely ask specifically about the demand for such options [12-17]. Usually, hypothetical scenarios of terminally ill persons are presented, and the participants are asked if euthanasia or assisted suicide is an appropriate option in a particular case [12-15]. In addition, some of the studies carry a particular risk of selection bias because of the topic’s controversial nature [12,14]. People who are unwilling to participate because of their fundamental beliefs may be underrepresented. Furthermore, proportionally high participation of ideological supporters of euthanasia and assisted suicide may influence the outcome through a self-selection bias in these voluntary surveys [14].

A novel approach for estimating public interests, awareness, and behavior is the analysis of big data obtained from internet search engines such as Google, Yahoo!, and Bing [18]. Recorded Google search volumes are publicly available in anonymized form on the Google Trends platform [19]. The data have already been used to measure public interest in cancer screening [20] and trend changes in the public awareness of palliative care after the implementation of new governmental policies [21] or the death of a celebrity [22]. Google Trends was also used in a variety of other medical and health-related disciplines [23]. It was used to predict and track infectious diseases such as influenza [24] and Lyme disease [25] with comparable accuracy to traditional methods and represents a valuable epidemiological tool.

Compared with traditional surveys that assess opinions, a Google Trends analysis is a useful tool for estimating information needs. Such trends serve as a proxy for the demand for health care services, information needs, and curiosity about health-related topics [23]. A survey across the European Union on internet use found that 58.71% (15,598/26,566) of the respondents used the internet to obtain health-related information [26]. Of those who tried to find specific information about medical treatments and procedures, 76.5% (2724/3561) conducted searches for themselves, and 30.3% (1079/3561) had done so for a family member [26]. A total of 40.51% (1119/2762) of those who rated their health as *bad* used the internet to obtain health-related information [26]. Hence, it is clear that the internet is an important source of information for Europeans, and search volumes represent the subjects of interest of a vast number of European Union citizens.

### Objective

In Germany and Austria, no data about the demand for euthanasia or assisted suicide are available to date, as no register or comparable data acquisition is possible because of the restriction and prohibition of the practices, respectively. Therefore, in this study, we hypothesize that internet search behavior might provide a novel approach to gaining essential insights into the general population’s needs and interests concerning palliative care, euthanasia, and end-of-life decisions. This study further aims to assess the influence of expanding care services and established governmental health care policies in both countries on the search behavior for each topic.

In addition, we chose Switzerland as a comparator as it is a (partly) German-speaking country and has already established practices for assisted suicide.

## Methods

### Data Collection

Google Trends data are available from January 2004 onward [19]. Therefore, we searched for relevant governmental policies, enacted or revised laws, and publicized health care strategies that emerged between January 2004 and December 2020 in each country (Germany, Austria, and Switzerland).

Every 6 years (starting in 2007), the EAPC publishes the *EAPC Atlas of Palliative Care in Europe* [27-29]. The report reviews the current status and availability of palliative care services for every European country. The available services per 100,000 citizens were obtained from each report to assess the development of palliative care over time.

Google gathers data on every search query performed on its search engine. The summarized data are publicly available in anonymous form on the Google Trends webpage [19]. Up to 5 search terms or topics can be simultaneously entered on the page. In contrast to search terms, search topics summarize several interrelated search terms and represent interest in a given topic more accurately. In addition, 4 filters can be applied: region, time, category, and search type. The output is the relative volume of queries for the search term or topic of interest to the total volume of queries in the selected region and period. The result is then scaled on a range of 0-100. Repeated search queries of a single user within a short time are not recorded so as to prevent intentional influence and enhance data quality [18].

In our study, we used three search term topics: *palliative care* plus *euthanasia* plus *advance health care directive*. All 3 terms were entered in the German language. The search topic *euthanasia* summarizes queries regarding assisted suicide and voluntary euthanasia. On January 25, 2021, we downloaded the data on the monthly relative search volume from January 2004 to December 2020 for Germany, Austria, and Switzerland. We used *all categories* and *web search* as additional filters. After downloading the data, we calculated the annual mean for each search topic and performed a trend analysis using the Joinpoint Regression Program [30]. Finally, we compared the trends in relative search volumes and the significant turning points (joinpoints) to the implementation or revision of government policy changes.

### Data Analysis

The Joinpoint Regression Program allows the analysis of trends over time and identifies apparent changes or turning points in the direction of a trend [30]. We calculated the yearly average from the monthly relative search volumes for each keyword and region and performed a joinpoint analysis using a log-linear Poisson regression model. Model selection was based on the Bayesian information criterion. This approach has already been established in previous studies investigating the impact of governmental policies on public awareness and internet search behavior [31].

## Results

### Legal Developments After 2004

Table 1 shows a timeline of public health strategies and passed or revised laws for each country. Before 2004, palliative care services were already established in all 3 countries but were still in their infancy [32]. At the turn of the millennium, the first health care policies concerning the care of terminally ill patients were implemented [33-35]. After that, in all 3 countries, continuous advancements were made concerning the financing, education, and implementation strategies for palliative care. Legal regulations about end-of-life decisions and advance health care declarations were first passed in 2006 in Austria [36], followed by Germany in 2009 [37] and Switzerland in 2013 [38]. The provision granted patients a higher degree of autonomy in their health care decisions. The legislature in Austria further discriminates between legally binding and nonbinding advance health care directives [36]. The latter aims to communicate the patient’s preferences for end-of-life care and allows the treating physician to reconsider a previously dismissed medical treatment or procedure depending on the situation, whereas the legally binding form provides a detailed and definite description of nontreatment decisions in predefined health conditions [36].

**Table 1:** Timeline of public health strategies and laws passed or revised after 2004 for Austria, Germany, and Switzerland.

Year	Austria	Germany	Switzerland
2004	Public health care strategy: concept of graded hospice and palliative care in Austria	Register of health care proxies	—^a^
2006	Law: advance health care directive (legally binding and nonbinding)	—	The Swiss Academy of Medical Sciences issues medical ethics guidelines for palliative care
2007	Law: health care proxy	Law: specialized ambulatory palliative care as a mandatory insurance benefit	—
2008	—	—	Novelty: adult guardianship law
2009	—	Law: advance health care directive	National strategy: Palliative Care 2010-2012
2012	—	—	National strategy: Palliative Care 2013-2015
2013	—	—	Law: advance health care directive
2015	A parliamentary commission of inquiry: “Dignity at the end of life” with 51 resolutions for financing, promoting, and establishing palliative care services	Law: hospice and palliative medicine statute; prohibition of services for assisted suicide	Establishment: National Platform for Palliative Care
2018	Novelty: adult guardianship law	—	—
2020	Constitutional court rescinds the prohibition of assisted suicide	Constitutional court rescinds the prohibition of services for assisted suicide	—

^a^No public health strategies and laws were passed or revised during this year.

Assisted suicide has been exempt from punishment since 1918 in Switzerland and 1751 in Germany, whereas it was explicitly prohibited in Austria in 1934 [39]. However, Germany restricted the commercial use of assisted suicide in 2015, meaning that neither a business-like organization nor a nonprofit institution could offer assistance in dying [4]. In 2020, the constitutional courts in Germany and Austria revised their restrictive laws and declared them unjust, paving the way for the legal practice of assisted suicide [4,5]. In both countries, legislators are currently forming meaningful regulations to prevent the duplicitous use of this practice. Voluntary euthanasia is still prohibited in all 3 countries [39].

### Development of Palliative Care Services

The results of the regulatory changes and public health strategies are regularly reported in the *EAPC Atlas of Palliative Care in Europe* [27-29]. In summary, the public accessibility of stationary, ambulatory, and voluntary services increased considerably in all 3 countries. Palliative care services per 100,000 citizens doubled from 2007 to 2019 from 0.6 to 2.2 in Austria, 0.4 to 1.1 in Germany, and 0.5 to 1.1 in Switzerland [27-29]. The results are summarized in Table 2.

**Table 2:** Development of palliative care services between 2007 and 2019 for each country.

Country	Palliative care services per 100,000 citizens		
	2007	2013	2019
Austria	0.6	1.4	2.2
Germany	0.4	0.7	1.1
Switzerland	0.5	0.8	1.1

### Internet Search Behavior

#### General Findings

**Figure 1:**
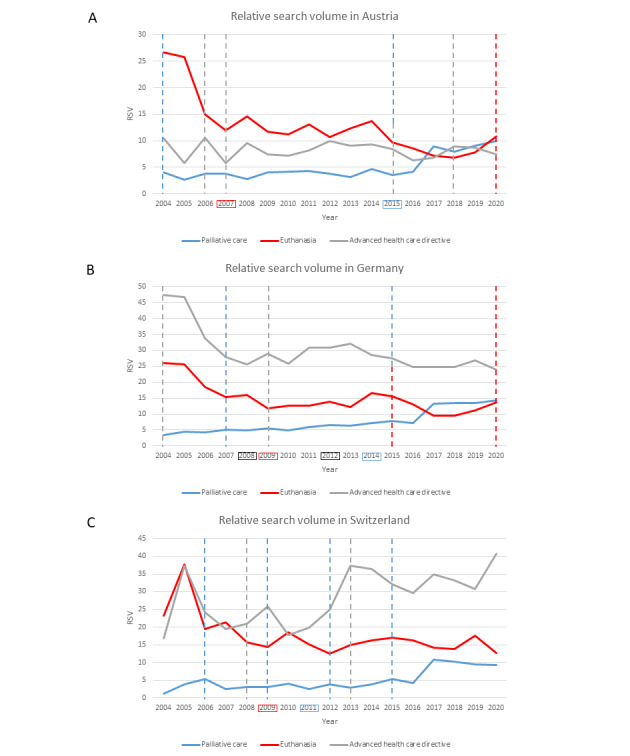
Trends in relative search volume for each country. Vertical dashed lines indicate passed or revised public health strategies or laws concerning palliative care, euthanasia, or advance health care directives. The colored frames indicate the years of significant trend changes according to the joinpoint analysis. RSV: relative search volume.

The search trends for each keyword and country are shown in Figure 1. In general, the search activity for palliative care increased over the studied period, whereas interest in euthanasia decreased in all 3 countries. The topic of advance health care directives showed mixed results. The greatest change in the average annual relative search volume for palliative care between 2004 and 2020 occurred in Switzerland, where there was an increase of 13.76% (95% CI 1.43-27.60; *P*=.03), followed by Germany with 9.15% (95% CI 6.12-12.27; *P*<.001) and then Austria (7.8%, 95% CI 2.96-12.88; *P*<.001).

Interest in euthanasia decreased in all 3 countries between 2004 and 2020. However, we found a significant decrease only in Switzerland, with a change of 4.15% (95% CI −8.09 to −0.04; *P*=.047) per year. For Germany and Austria, the relative search volume decreased by 4.13% (95% CI −11.09 to 3.38; *P*=.27) and 6.07% (95% CI −12.78 to 1.15; *P*=.10), respectively.

We found different results for the search behavior of advance health care directives in each country. Although the relative search volume remained at a consistent level in Austria, with an insignificant decrease of 0.16% (95% CI −2.27 to 1.99; *P*=.87) across the study period, the volume significantly decreased by 2.85% (95% CI −4.32 to −1.35; *P*<.001) in Germany and significantly increased by 3.66% (95% CI 1.18-6.21; *P*<.001) in Switzerland.

#### Austria

The joinpoint analysis revealed significant trend changes in the search terms’ trajectories, which are presented in Table 3. We recorded joinpoints for the interest topics of palliative care and euthanasia; however, none were found for advance health care directives. In 2006 and 2007, two new laws regulating advance health care directives and the appointment of health care proxies were enacted by the Austrian parliament. Following these enactments, the relative search volume for euthanasia dropped by 24.48% (95% CI −39.20 to −6.19). Afterward, 2 periods with an insignificant but continuous reduction occurred, although this trend was more prominent from 2014 to 2018 than from 2007 to 2014 (Table 3). Toward the end of the study period in 2020, we observed a positive trend, although the difference was not significant (Table 3). In 2015, the parliamentary commission of inquiry regarding *dignity at the end of life* adopted 51 resolutions to promote and fund palliative care, followed by a 22.69% (95% CI 7.05-40.61; *P*=.01) increase in the search interest in palliative care. Until then, there had been a positive but small slope (Table 3). Although it fluctuated over the whole study period, the search trend for advance health care directives remained at a steady level without significant trend changes between 2004 and 2020 (Table 3).

**Table 3:** Trend periods and annual percentage of change in the search volume for each term in Austria.

Trend periods		Annual change (95% CI; %)	*P* value
**Palliative care**			
	2004-2015	1.65 (−2.44 to 5.91)	.40
	2015-2020	22.69 (7.05 to 40.61)	.01
**Euthanasia**			
	2004-2007	−24.48 (−39.20 to −6.19)	.02
	2007-2014	−0.42 (−7.46 to 7.15)	.89
	2014-2018	−15.02 (−31.59 to 5.55)	.12
	2018-2020	29.74 (−15.91 to 100.17)	.19
**Advance health care directive**			
	2004-2020	−0.16 (−2.27 to 1.99)	.87

#### Germany

The search trends in Austria and Germany were similar; however, the latter had joinpoints in all 3 keywords. The results are summarized in Table 3. With the legal regulation of advance health care directives in 2009, the relative search volume for euthanasia dropped by 14.39% (95% CI −21.80 to −7.50; *P*<.001). After a slight positive trend, a negative slope appeared in 2015, followed by a positive trend in 2018. However, none of these changes were significant (Table 4). Within the relative search volume trend for palliative care, one joinpoint was determined in 2014, dividing the overall period into 2 segments of significant positive slopes. Although in the first period the interest grew continuously by 6.12% (95% CI 2.93-9.40; *P*<.001), a sudden rise of 14.39% (95% CI 7.06-22.23; *P*<.001) appeared in 2014 after the enactment of a law regulating hospice and palliative care accessibility. Interestingly, the search trend for advance health care directives followed a pattern comparable with the trend for euthanasia, with a drop of 15.42% (95% CI −22.03 to −8.25; *P*<.001) in 2008. Afterward, the search activity grew between 2008 and 2012 but became negative thereafter (Table 4).

**Table 4:** Trend periods and annual percentage of change in the search term for each term in Germany.

Trend periods		Annual change (95% CI; %)	*P* value
**Palliative care**			
	2004-2014	6.12 (2.93 to 9.40)	<.001
	2014-2020	14.39 (7.06 to 22.23)	<.001
**Euthanasia**			
	2004-2009	−14.95 (−21.80 to −7.50)	<.001
	2009-2015	4.13 (−4.26 to 13.26)	.28
	2015-2018	−16.83 (−42.88 to 21.08)	.27
	2018-2020	24.97 (−14.17 to 81.95)	.20
**Advance health care directive**			
	2004-2008	−15.42 (−22.03 to −8.25)	<.001
	2008-2012	5.19 (−7.49 to 19.62)	.39
	2012-2020	−3.30 (−5.98 to −0.55)	.01

#### Switzerland

In Switzerland, similar trends in search activity, presented in Table 5, were found. The adult guardianship law was amended in 2008, and it included the regulation of advance health care directives. This amendment was followed by a decrease of 11.75% (95% CI −22.09 to −0.04; *P*=.049) in the relative volume of searches for euthanasia between 2004 and 2009. In the following years, the trend remained stable and slightly negative (Table 5). However, this result is likely biased by the extraordinary peak in the relative search volume in 2005. At that time, the broad public debate about euthanasia and *suicide tourism* coincided with a higher search activity [40].

**Table 5:** Trend periods and annual percentage of change in the search volume for each term in Switzerland.

Trend periods		Annual change (95% CI; %)	*P* value
**Palliative care**			
	2004-2006	73.35 (−21.41 to 282.38)	.15
	2006-2011	−9.44 (−29.48 to 16.30)	.39
	2011-2020	17.59 (9.40 to 26.40)	<.001
**Euthanasia**			
	2004-2009	−11.75 (−22.09 to −0.04)	.049
	2009-2020	−0.49 (−4.15 to 3.32)	.78
**Advance health care directive**			
	2004-2020	3.66 (1.18 to 6.21)	<.001

Nevertheless, after 2009 (the calculated joinpoint), no likewise peak was observed, and the overall trend followed a negative slope (Table 5). For palliative care, the search activity increased significantly by 17.59% (95% CI 9.40-26.40; *P*<.001) from 2011 onward after the government proposed its first national strategy for promoting the implementation of palliative care. Interest in advance health care directives increased significantly by 3.66% (95% CI 1.18-6.21; *P*<.001) across the study period.

## Discussion

### Principal Findings

Our study results show that between 2004 and 2020, substantial legal and public health efforts were made to promote patients’ autonomy in regulating end-of-life decisions and develop palliative care services in Austria, Germany, and Switzerland, as summarized in Table 1. According to our analysis, a reduction in search activity for topics related to euthanasia ensued. At the same time, interest in palliative care in all 3 countries increased. Our results support previous findings regarding why individuals consider or eventually choose voluntary death. The fear of losing control over medical decisions and being helpless against a system capable of unnecessarily prolonging a life of suffering puts individuals under pressure to take action themselves [41]. Euthanasia, assisted suicide, and advance health care directives grant individuals a high level of autonomy and self-control, especially at the end of their life. Precise regulations for advance health care directives may have provided a viable alternative option to assisted suicide. For example, the provision of alternatives decreased interest in euthanasia, according to our results. Of note is the significant increase in the search interest in advance health care directives in Switzerland, even though assisted suicide is available. Specifically, we found significant trend changes that aligned with specific governmental actions.

We found that public awareness of palliative care increased with the realization of public health strategies and the enactment of laws to regulate funding, education, accessibility, and insurance coverage related to palliative care. A previous study using Google Trends data from the United States showed similar results, with increased public awareness for palliative care from January 2005 to December 2015 [21]. The authors also suggest a correlation between this increase in search queries and changes in insurance providers’ policies promoting advance care planning and the growth in nationwide palliative care service provision [21]. This result underlines the importance of government efforts to strengthen public awareness and promote access to palliative care services for those who need them. It is important to mention that interest in palliative care also increased in Switzerland, a country with established practices for assisted suicide. However, the government actions concerning palliative care did not align with the trend changes in the search activity for euthanasia, suggesting that there were other factors with a more significant impact on the decreased interest in assisted suicide and death on demand, such as legal regulations concerning the promotion of autonomy at the end of life. The embedment of advance health care directives and the legal appointment of health care proxies into a legal framework was followed by a significant drop in search queries for terms related to euthanasia and assisted suicide.

Before legal regulations were put in place, advance health care directives were a gray area and were theoretically not legally binding, allowing them to be overruled by caregivers and medical staff without facing consequences [42]. The formulation of a sound legal framework eliminated this uncertainty for patients and has potentially led to a higher sense of autonomy, which is in a complex relationship with perceived dignity and personal identity [10]. The desire to maintain a sense of control and autonomy also includes the desire to control different aspects of one’s dying process but not necessarily wishing to hasten death actively [43]. However, situations threatening autonomy may be experienced as an undermining of one’s dignity, evoking existential fears of patients that may produce a sense of urgency to take self-action [43]. On the other hand, promoting autonomy with the provision of advance care planning leads to higher emotional and mental well-being [44] and quality of life [45] of patients with progressive life-limiting diseases. It appears that the legal regulations of advance health care directives secured the wish to maintain control over the dying process and led to the decreased necessity of seeking information about other opportunities, such as traveling to other countries for assisted suicide.

The significant increase in popularity of search terms related to advance health care directives in Switzerland, the only studied country with established legal practices of assisted suicide, supports the hypothesis that they are a viable alternative option. However, this trend showed different results in the other 2 countries compared with that in Switzerland. At the beginning of the study period, in Germany, the high search volume might have resulted from a high degree of uncertainty about the legal options available, leading to increased search activity to obtain information about the requirements for advance health care directives. Formulating clear regulations could have led to a drop in the queries as it was easier to obtain precise details about such practices. In Austria, the search volume for advance health care directives did not change considerably during the study period. This raises the question of which factors may have contributed to this trend. Although the new legal framework provides clear definitions for legally binding directives and grants a high degree of self-determination through its binding character, Austrians face a cost barrier of up to €500 (US $584.50) for the certifying notary [46]. This is approximately one-third of the average middle-class salary and hinders low-income Austrians from accessing this option [46]. In practice, patients use legally binding directives less frequently than nonlegally binding directives, which require neither notarial certification nor informed consent or discussion with a physician [47]. Therefore, patients may not seek as much information about how to arrange a legally binding directive or may discuss potentially confusing options primarily with their physicians.

Given the current developments in Germany and Austria, which tend to legalize assisted suicide practices, palliative care services could be involved in the decision-making process to find the best solution for each patient. Professional palliative caregivers are experienced in discussing sensitive topics such as end-of-life decisions and can inform patients about the different options in an evidence-based manner. Recent literature reviews have highlighted the benefits of early palliative care implementation in oncology, including an increase in the number of stipulated advance health care directives [48]. Therefore, palliative care may serve as a filter for patients with a genuinely intrinsic wish to hasten death actively and guide them in a meaningful and supportive way [8]. Evaluating the underlying reasoning, fears, and perhaps misunderstandings of individuals about end-of-life care is crucial in preventing the misuse of euthanasia and assisted suicide [49-52]. With the legalization of assisted suicide, it would be reasonable to simultaneously increase the accessibility of palliative care and extend its early integration into the treatment of terminally ill patients.

### Limitations

Some limitations of this study need to be discussed. First, this is a correlative retrospective study, and hence, it does not allow for definite conclusions about causal relationships between the enactment of policies and the investigated search trends. However, we tried to minimize the potential bias by comparing the trends in 3 comparable countries and were able to show similar results, even in Switzerland, which has notably liberal regulations concerning the practice of assisted suicide.

Second, Google Trends does not provide absolute numbers and therefore lacks transparency to some extent. Researchers can rely only on the process description that Google publishes on its webpage concerning the handling of its data [19]. Of note are the preventive measures taken against bias by limiting the number of queries per user within a specific time. With this, active manipulation and repeated search queries (eg, during a literature search) did not influence the overall search volume and therefore represented the search activity within a region more accurately.

Third, we used search topics instead of definite search terms to limit the chances of choosing a less commonly used search term. However, this option may have also included nonspecific search terms. The most popular search terms are still included in the output of a trend query, and in our case, these queries predominantly showed specific terms related to the research question in this study. The output (in German) for each country and each search topic are presented in Tables S1-S3 in Multimedia Appendix 1.

Fourth, the trend changes might have been influenced by factors other than legal regulations and health care policies. For example, through the constant improvement of disease-specific and supportive therapies over the past two decades, the disease burden of many life-limiting diseases decreased while simultaneously focusing on enhancing patients’ quality of life [53].

### Implications

Our study provides novel and essential information about the demands and interests of the residents of Austria, Germany, and Switzerland concerning palliative care, euthanasia, assisted suicide, and end-of-life decisions. In particular, policy makers may use these findings to target potential weaknesses within current regulations (eg, the cost barrier for advance health care directives in Austria). Governmental promotion of the early implementation of palliative care into life-threatening disease trajectories may be another meaningful action. Experts in palliative care can sensitize patients to arranging advance health care directives, inform them about the different options at the end of life, and resolve misunderstandings.

### Conclusions

Our study shows, for the first time, that governmental regulations, precise legal definitions, and broader access to advance health care directives might reduce interest in euthanasia, suggesting that loss of control is one of the predominant factors involved in the desire to hasten death. Early palliative care can guide patients through the necessary decision-making processes from the point of the diagnosis of a life-threatening disease to their death and provide advance care planning. After all, it is and has always been a core principle of palliative care to promote and value the autonomy of patients.

## Appendix

Multimedia Appendix 1 Most popular search terms.

## Abbreviations

EAPC: European Association of Palliative Care
