# Supplementary material for: Trends and Patterns in the Public Awareness of Palliative Care, Euthanasia, and End-of-Life Decisions in 3 Central European Countries Using Big Data Analysis From Google: Retrospective Analysis
Source: J Med Internet Res. 2021 Sep 20;23(9):e28635. doi: 10.2196/28635 (PMC8491122; doi:10.2196/28635)
Supplement: Multimedia Appendix 1 [file jmir_v23i9e28635_app1.doc]

|  | **German search term** | **English translation** | **RSV** |
| --- | --- | --- | --- |
|  |  |  |  |
| **Search topic “palliative care”** |  |  |  |
|  | palliativ | palliative | 100 |
|  | palliativmedizin | Palliative medicine | 67 |
|  | hospiz | hospice | 8 |
|  | paliativ | misspelled “palliative” | 5 |
|  | palliativstation | palliative care ward | 5 |
|  | paleativ | misspelled “palliative” | 3 |
|  | geriatrie | Geriatric medicine | 3 |
|  | palliativ bedeutung | palliative meaning | 1 |
| **Search topic “euthanasia”** |  |  |  |
|  | sterbehilfe | euthanasia | 100 |
|  | euthanasie | euthanasia | 50 |
|  | sterbehilfe österreich | euthanasia Austria | 16 |
|  | euthanasia | euthanasia | 16 |
|  | aktive sterbehilfe | active euthanasia | 10 |
|  | sterbehilfe in österreich | euthanasia in Austria | 6 |
|  | sterbehilfe deutschland | euthanasia Germany | 4 |
|  | sterbehilfe niederlande | euthanasia netherlands | 4 |
|  | passive sterbehilfe | passive euthanasia | 3 |
|  | sterbehilfe deutschland urteil | euthanasia Germany verdict | <1 |
|  | sterbehilfe schweiz kosten | euthanasia Switzerland costs | <1 |
| **Search topic “advance healthcare directive” (AHCD)** |  |  |  |
|  | patientenverfügung | AHCD | 100 |
|  | patientenverfügung österreich | AHCD Austria | 26 |
|  | patientenverfügung formular | AHCD form | 11 |
|  | vorsorgevollmacht | health care proxy | 7 |
|  | patientenverfügung wien | AHCD Vienna | 6 |
|  | patientenverfügung kosten | AHCD costs | 4 |
|  | patientenverfügung muster | AHCD model | 4 |
|  | patientenverfügung österreich formular | AHCD Austria form | 3 |
|  | dnr medizin | DNR medicine | 1 |

Table S1: Top search terms in Austria between 2004 and 2020

Table S2: Top search terms in Germany between 2004 and 2020

|  | **German search term** | **English translation** | **RSV** |
| --- | --- | --- | --- |
|  |  |  |  |
| **Search topic “palliative care”** |  |  |  |
|  | palliativmedizin | palliative medicine | 100 |
|  | palliativ | palliative | 91 |
|  | hospiz | hospice | 8 |
|  | paliativ | misspelled “palliative” | 6 |
|  | palliativstation | palliative care ward | 6 |
|  | palliative | misspelled “palliative” | 5 |
|  | palliativ medizin | palliative medicine | 3 |
|  | palliativmediziner | palliative care physician | 3 |
|  | was ist palliativmedizin | what is palliative medicine | 3 |
|  | paleativ | misspelled “palliative” | 3 |
|  | palliativpflege | palliative nursing | 2 |
|  | sterbehilfe | euthanasia | 2 |
|  | palliativ care | palliative care | 2 |
|  | sapv | specialiced ambulant palliative care | 2 |
|  | onkologie | oncology | 2 |
|  | palliativ definition | palliative definition | 2 |
|  | palliativmedizin definition | palliative medicine definition | 2 |
|  | schmerztherapie | analgesia | 2 |
|  | weiterbildung palliativmedizin | advanced training palliative medicine | 2 |
|  | palliativversorgung | palliative care service | 1 |
|  | palliativ bedeutung | palliative meaning | 1 |
|  | geriatrie | geriatric medicine | 1 |
|  | was bedeutet palliativ | what does palliative mean | 1 |
|  | palliative care | palliative care | 1 |
|  | was ist palliativ | what is palliative | 1 |
| **Search topic “euthanasia”** |  |  |  |
|  | sterbehilfe | euthanasia | 100 |
|  | euthanasie | euthanasia | 37 |
|  | deutschland sterbehilfe | Germany euthanasia | 12 |
|  | aktive sterbehilfe | active euthanasia | 12 |
|  | sterbehilfe schweiz | euthanasia Switzerland | 11 |
|  | euthanasia | euthanasia | 7 |
|  | sterbehilfe in deutschland | euthanasia in Germany | 6 |
|  | passive sterbehilfe | passive euthanasia | 6 |
|  | sterbehilfe contra | euthanasia contra | 3 |
|  | eutanasia | misspelled “euthanasia” | 3 |
|  | sterbehilfe niederlande | euthanasia Netherlands | 3 |
|  | sterbehilfe pro contra | euthanasia pro contra | 3 |
|  | sterben | dying | 3 |
|  | sterbehilfe definition | euthanasia definition | 2 |
|  | indirekte sterbehilfe | indirect euthanasia | 2 |
|  | suizid | suicide | 2 |
|  | dignitas | dignitas | 2 |
|  | belgien sterbehilfe | Belgium euthanasia | 2 |
|  | eutanasie | misspelled “euthanasia” | 2 |
|  | aktive passive sterbehilfe | active passive euthanasia | 2 |
|  | sterbehilfe holland | euthanasia Holland | 2 |
|  | dignitas sterbehilfe | dignitas euthanasia | 2 |
|  | sterbehilfe erlaubt | euthanasia permitted | 2 |
|  | euthanasie definition | euthanasia definition | 2 |
|  | sterbehilfe in der schweiz | euthanasia in Switzerland | 2 |
| **Search topic “ADHC”** |  |  |  |
|  | patientenverfügung | ADHC | 100 |
|  | formular | from | 14 |
|  | patientenverfügung formular | ADHC form | 13 |
|  | vorsorgevollmacht | health care proxy | 12 |
|  | vorsorgevollmacht patientenverfügung | health care procy ADHC | 11 |
|  | patientenverfügung vordruck | ADHC preprint | 8 |
|  | patientenverfügung kostenlos | ADHC free | 7 |
|  | patientenverfügung muster | ADHC model | 5 |
|  | vorlage patientenverfÜgung | template ADHC | 4 |
|  | patientenverfügung pdf | ADHC pdf | 4 |
|  | patientenverfügung ausdrucken | ADHC print out | 3 |
|  | patientenverfügung formular kostenlos | ADHC free from | 3 |
|  | patientenverfügung zum ausdrucken | printable ADHC | 3 |
|  | vorsorgevollmacht und patientenverfügung | health care proxy and ADHC | 3 |
|  | bundesministerium patientenverfügung | federal ministry ADHC | 2 |
|  | bundesministerium | federal ministry | 2 |
|  | patientenverfügung download | ADHC download | 2 |
|  | vorsorgevollmacht formular | health care proxy form | 2 |
|  | betreuungsverfügung | health care proxy | 2 |
|  | testament | will | 2 |
|  | patientenverfügung vollmacht | ADHC warrant | 2 |
|  | vollmacht | warrant | 2 |
|  | bundesministerium justiz patientenverfügung | federal ministry justice ADHC | 2 |
|  | Ärztekammer patientenverfügung | medical association ADHC | 2 |
|  | Ärztekammer | medical association | 1 |

Table S3: Top search terms in Switzerland between 2004 and 2020

|  | **German search term** | **English translation** | **RSV** |
| --- | --- | --- | --- |
|  |  |  |  |
| **Search topic “palliative care”** |  |  |  |
|  | palliativ | palliative | 100 |
|  | palliativmedizin | palliative medicine | 64 |
|  | paliativ | misspelled “palliative” | 11 |
| **Search topic “euthanasia”** |  |  |  |
|  | sterbehilfe | euthanasia | 100 |
|  | euthanasie | euthanasia | 55 |
|  | sterbehilfe schweiz | euthanasia Switzerland | 32 |
|  | exit | exit | 26 |
|  | exit sterbehilfe | exit euthanasia | 22 |
|  | euthanasia | euthanasia | 21 |
|  | eutanasia | misspelled “euthanasia” | 14 |
|  | aktive sterbehilfe | active euthanasia | 10 |
|  | dignitas | dignitas | 6 |
|  | sterbehilfe deutschland | euthanasia Germany | 2 |
| **Search topic “ADHC”** |  |  |  |
|  | patientenverfügung | ADHC | 100 |
|  | fmh | Swiss Medical Association | 22 |
|  | fmh patientenverfügung | Swiss Medical Association AHCD | 21 |
|  | patientenverfügung schweiz | AHCD Switzerland | 19 |
|  | formular patientenverfügung | form AHCD | 12 |
|  | directives anticipées | AHCD | 8 |
|  | vorsorgeauftrag | health care proxy | 5 |
|  | patientenverfügung muster | AHCD model | 4 |
|  | srk | swiss red cross | 4 |
|  | patientenverfügung srk | AHCD swiss red cross | 4 |
|  | pro senectute | AHCD | 4 |
|  | vorlage patientenverfügung | template AHCD | 4 |
|  | patientenverfügung pro senectute | AHCD | 4 |
|  | patientenverfügung kostenlos | AHCD free | 3 |
|  | patientenverfügung formular schweiz | AHCD form Switzerland | 3 |
|  | exit patientenverfügung | exit AHCD | 2 |
|  | fmh directives anticipées | Swiss Medical Association AHCD | 2 |
|  | patienten | patients | 2 |
|  | patientenverfügung ch | AHCD ch | 2 |
|  | patientenverfügung pdf | AHCD pdf | 2 |
|  | caritas | caritas | 2 |
|  | samw patientenverfügung | Swiss Academies of Arts and Sciences AHCD | 1 |
|  | patientenverfügung vordruck | AHCD preprint | 1 |
|  | patientenverfügung kurzversion | AHCD short form | 1 |
|  | vorsorgeauftrag muster | healthy care proxy model | 1 |
